# Complete genomic sequence of a *Marinobacter* species, a potential polyethylene degrader isolated from surface seawater

**DOI:** 10.1128/mra.00616-24

**Published:** 2024-08-20

**Authors:** Ryo Iizuka, Sotaro Uemura

**Affiliations:** 1 Department of Biological Sciences, Graduate School of Science, The University of Tokyo, Tokyo, Japan; 2 Core Research for Evolutional Science and Technology (CREST), Japan Science and Technology Agency, Tokyo, Japan; Montana State University, Bozeman, Montana, USA

**Keywords:** *Marinobacter*, polyethylene, alkane

## Abstract

The bacterium *Marinobacter* sp. RI1 was isolated from surface seawater through an enrichment culture using low-density polyethylene as the sole carbon source. Herein, we report its complete genomic sequence. Genomic annotation revealed that the strain harbors the genes encoding enzymes involved in alkane degradation, thus supporting polyethylene degradation.

## ANNOUNCEMENT

*Marinobacter* species are frequently found in hydrocarbon-contaminated marine environments, suggesting an important role in countering hydrocarbon pollution (1). Here, we present the complete genomic sequence of *Marinobacter* sp. RI1, an isolate from surface seawater with potential polyethylene degradation capability.

Surface seawater was collected at Odaiba Beach, Tokyo, Japan (35°37′50.3″N, 139°46′30.2″E) on 16 August 2023 and was inoculated into artificial seawater (GEX, Osaka, Japan) containing 0.1% NH_4_Cl and an untreated low-density polyethylene film (10 × 10 mm; NPS-0.5, KENIS, Tokyo, Japan). After 139 days of incubation at 25°C, the film surface was scraped with an inoculation loop, and bacterial cells were streaked onto an agar plate containing artificial seawater medium (2, 3). The plate was incubated for 2 days at 25°C, resulting in the isolation of strain RI1. Colony PCR was performed using the primers 27F (5′-AGAGTTTGATCMTGGCTCAG-3′) and 1492R (5′-TACGGYTACCTTGTTACGACTT-3′) to amplify the 16S rRNA gene. The sequence obtained (LC819396.1) had the highest similarity (99.93%) with that of *Marinobacter nauticus* DSM 50418^T^ (AB021372.1) in the EzBioCloud database (4, 5). The strain was cultured aerobically in artificial seawater medium overnight at 25°C for genomic sequencing. Genomic DNA was extracted using the Genomic-tip 20/G (Qiagen), followed by purification using the Short Read Eliminator (PacBio) and DNA Clean Beads (MGI Tech Co., Ltd.). Genomic DNA was sheared into 10–20 kbp fragments using a g-TUBE (Covaris). A SMRTbell library was prepared using SMRTbell gDNA Sample Amplification Kit (PacBio) and SMRTbell Express Template Prep Kit 2.0 (PacBio), and then bound to DNA polymerase using Revio Polymerase Kit (PacBio). Sequencing was performed using the Revio system (PacBio). SMRT Link (v13.0.0.207600) (PacBio) was used to trim adapter sequences from the sequencing reads. The generated circular consensus sequences with average base quality values of <20 were removed to obtain HiFi reads. These reads were filtered using lima (v2.7.1) (https://github.com/pacificbiosciences/barcoding) and pbmarkdup (v1.0.3) (https://github.com/PacificBiosciences/pbmarkdup) to remove the ultra-low PCR adapters and PCR duplicates, respectively. Filtlong (v0.2.1) (https://github.com/rrwick/Filtlong) was used to exclude short HiFi reads (<1,000 bp). Using Flye (v2.9.2-b1786) (6), the remaining reads were assembled, and the overlapping ends on a circular contig were removed. Automatic annotation was performed using DFAST v1.3.1 (7, 8). Default settings were used for all software.

The RI1 genome was found to comprise a circular chromosome of 3,846,721 bp, harboring 3,466 protein-coding sequences, nine rRNA genes, and 51 tRNA genes (Table 1). Phylogenetic analysis suggested that the closest relative was *M. nauticus* DSM 50418^T^ (GCF_003634635.1) with a pairwise average nucleotide identity of 97.7%, implying that the strain is a novel strain of *M. nauticus*. Genomic annotation revealed the presence of genes encoding key enzymes in alkane degradation pathways, including alkane 1-monooxygenases (MspRI1_13460 and MspRI1_15020), cytochrome P450 alkane hydroxylase (CYP153; MspRI1_05720), and flavin-binding monooxygenase (AlmA; MspRI1_05860) (9–11), which are likely responsible for polyethylene catabolism (12, 13). This genomic information provides insights into the genetic basis of the catabolic pathways for polyethylene degradation in this strain.

**TABLE 1:** Genomic characteristics of the sequenced isolate

	Strain RI1
BioSample accession no.	SAMD00787955
No. of reads	40,328
Read N50 (bp)	6,570
Sum of length (bp)	248,538,631
SRA accession no.	DRR568656
Assembly results	
No. of contigs	1
Length (bp)	3,846,721
GC content (%)	57.6
Average read depth (×)	65
No. of protein-coding sequences	3,466
No. of rRNAs	9
No. of tRNAs	51
GenBank accession no.	AP031605.1

## Data Availability

RI1 is associated with the BioProject accession number PRJDB18161. The accession numbers are listed in Table 1.
